# The Lunar Environment Heliophysics X-ray Imager (LEXI) Mission

**DOI:** 10.1007/s11214-024-01063-4

**Published:** 2024-05-14

**Authors:** B. M. Walsh, K. D. Kuntz, S. Busk, T. Cameron, D. Chornay, A. Chuchra, M. R. Collier, C. Connor, H. K. Connor, T. E. Cravens, N. Dobson, M. Galeazzi, H. Kim, J. Kujawski, C. K. Paw U, F. S. Porter, V. Naldoza, R. Nutter, R. Qudsi, D. G. Sibeck, S. Sembay, M. Shoemaker, K. Simms, N. E. Thomas, E. Atz, G. Winkert

**Affiliations:** 1 Center for Space Physics, Boston University, Boston, 02215 MA USA; 2 The Henry A. Rowland Department of Physics and Astronomy, Johns Hopkins University, Baltimore, 21218 MD USA; 3 NASA, Goddard Space Flight Center, Greenbelt, 20771 MD USA; 4 Thermac LLC, Annapolis, 21401 MD USA; 5 Department of Physics and Astronomy, University of Kansas, Lawrence, 66045 KS USA; 6 Department of Physics, University of Miami, Miami, 33146 FL USA; 7 Space Research Institute, Austrian Academy of Sciences, Graz, Austria; 8 Brandywine Photonics, College Station, 77845 TX USA; 9 School of Physics and Astronomy, University of Leicester, Leicester, UK; 10 Marshall Space Flight Center, NASA, Huntsville, 35808 AL USA

**Keywords:** Magnetopause, X-ray imaging, Reconnection, Lunar

## Abstract

The Lunar Environment heliospheric X-ray Imager (LEXI) is a wide field-of-view soft X-ray telescope developed to study solar wind-magnetosphere coupling. LEXI is part of the Blue Ghost 1 mission comprised of 10 payloads to be deployed on the lunar surface. LEXI monitors the dayside magnetopause position and shape as a function of time by observing soft X-rays (0.1–2 keV) emitted from solar wind charge-exchange between exospheric neutrals and high charge-state solar wind plasma in the dayside magnetosheath. Measurements of the shape and position of the magnetopause are used to test temporal models of meso- and macro-scale magnetic reconnection. To image the boundary, LEXI employs lobster-eye optics to focus X-rays to a microchannel plate detector with a 9.1$^{\circ }\times 9.1^{\circ }$ field of view.

## Introduction

Global imaging of Earth’s space environment offers great potential to answer outstanding questions in heliophysics and fundamental sciences. Starting with lunar surface-based EUV observations of the global terrestrial exosphere by the Apollo 16 astronauts (Carruthers et al. 1976), space-based imaging has both illuminated critical elements and answered many system-level questions in the complex coupled magnetosphere-ionosphere-thermosphere-exosphere system. A necessary component to advancing science through imaging is obtaining a sufficient field-of-view (FOV) for the desired science target. To address system-level questions, this often requires an observer to be outside of the subject of study. LEXI obtains this critical vantage outside of the magnetosphere by observing from the lunar surface.

**Fig. 1:**
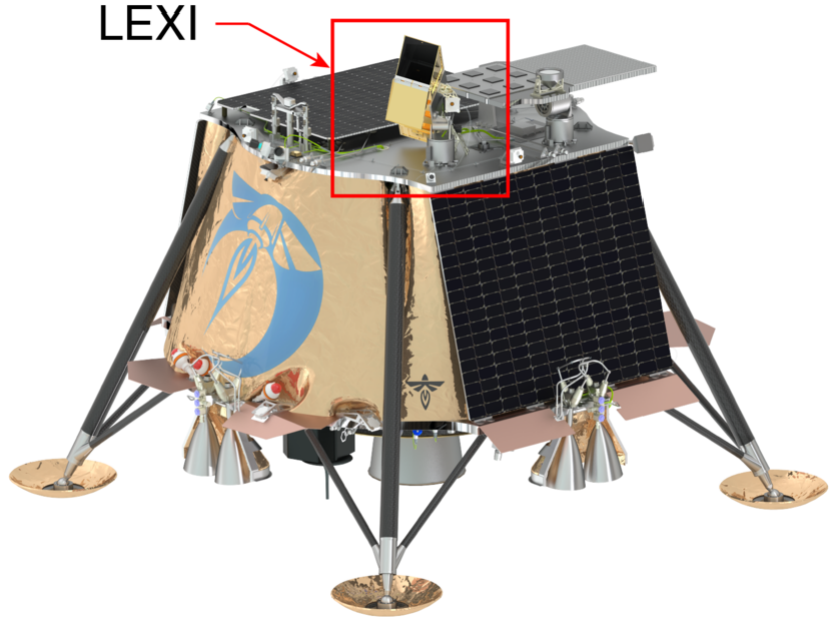
Lunar lander with LEXI mounted on a motional stage on the top deck

LEXI is hosted on the Blue Ghost 1 Lander developed by Firefly Aerospace to be operated on the lunar surface in 2024. LEXI is one of ten payloads on the lander as part of the NASA Commercial Lunar Payloads Service (CLPS) program, task order 19D. The diverse set of payloads target a range of science and technology objectives. Figure 1 presents a model of the lander showing LEXI’s position mounted on the top deck to enable an unobstructed FOV and to separate the payload from vibrations and lunar dust generated by active surface experiments on the underside of the lander.

Blue Ghost 1 will land and operate from the lunar surface at a horse-shoe shaped feature, Mons Latreille within Mare Crisium at 18.5604^∘^ N, 61.8071^∘^ E. The landing site was selected based on its low slope ($<2^{\circ}$ over 4 m), absence of magnetic anomalies, and low rock abundance (0.002 diviner rock abundance), providing a flat landing surface. From this geometry LEXI will be able to measure X-ray emissions continuously origionating from Earth’s magnetosheath for 6.5 days. Transit from Earth to the moon will take approximately 40 days depending on the launch date. During this transit period LEXI will perform periodic health and calibration checks but will not take science measurements.

## Science Objectives and Background

### Soft X-ray Imaging

The LEXI mission images photons resulting from solar wind charge-exchange. Anywhere an ion encounters a neutral atom charge-exchange can occur. Within the heliosphere, flowing plasma in the solar wind routinely encounters environments of dense neutrals surrounding comets, moons, and planets. In these regions, high charge state heavy ions from the solar wind charge-exchange with neutrals from the body’s exosphere. One sample interaction is given by

1 $$ \mathrm{O}^{7+} + \mathrm{H} \rightarrow \mathrm{O}^{6+*} + \mathrm{H}^{+} $$

and then

2 $$ \mathrm{O}^{6+*} \rightarrow \mathrm{O}^{6+} + \nu $$

where the asterisk denotes that $O^{6+}$ is in an excited state. In this interaction an electron is transferred from the neutral hydrogen to the high charge state ion in an excited state. The newly acquired electron rapidly transitions to a lower energy state, releasing a soft X-ray photon ($\nu $), typically with energies from 0.1–2.0 keV. Emission lines have been identified from a number of high charge state species charge-exchanging in the solar system including C^6+^, O^7+^, O^8+^, Mg^12+^, and Fe^13+^ (Lisse et al. 2001; Snowden et al. 2004; Fujimoto et al. 2007; Carter and Sembay 2008; Ezoe et al. 2010). High charge state ions such as O^7+^ are created in the million degree solar corona which is the source of the solar wind.

3 $$ F \, [\mathrm{photons}, \mathrm{cm}^{-2}\,\mathrm{s}^{-1}\,\mathrm{sr}^{-1}] = \int _{0}^{\infty} (n_{n} n_{sw} v_{rel} \langle \sigma \rangle b f) \frac{d\Omega}{4\pi} dl $$

The total emission ($F$) an observer would measure from this X-ray generation mechanism is a line-of-sight integration. Equation (3) presents a description of this integrated emission where $n_{n}$ is the neutral density, $n_{sw}$ is the solar wind proton density, $\langle \sigma \rangle $ is the mean interaction cross-section, $f$ is the ratio of the density of the ion producing the line to the proton density, and $b$ is the fraction of interactions that produce a photon in the line of interest. The relative velocity of the ions and the neutrals, $v_{rel}$, is given by

4 $$ v_{rel}=\sqrt{(v_{b}^{2}+v_{therm}^{2})} $$

where $v_{therm}\sim 3{k_{b}}T/m_{p}$ for the solar wind, $k_{b}$ is Boltzmann’s constant, and $v_{b}$ is the bulk flow velocity. Kuntz et al. (2015) provides detailed calculation and simulation of soft X-ray production with solar wind plasma and hydrogen.

Regions such as Earth’s magnetosheath and cusps, where dense solar wind plasma is collocated with dense exospheric neutrals from Earth (Robertson and Cravens 2003; Walsh et al. 2016a), are bright emitters of soft X-rays, as demonstrated by narrow (<1 deg^2^) field-of-view astrophysics missions (Cravens et al. 2001; Snowden et al. 2004; Fujimoto et al. 2007; Carter et al. 2011). The magnetopause boundary delineates a region of high solar wind density (magnetosheath) from one of low density (magnetosphere). As such, the boundary is readily identifiable through a strong gradient in soft X-ray flux (Walsh et al. 2016b; Sibeck et al. 2018; Sun et al. 2019; Connor et al. 2021; Kuntz et al. 2022; Ng et al. 2023).

### Dayside Solar Wind-Magnetosphere Interaction

Earth’s magnetosphere and ionosphere are driven, during quiet times and disturbed, through its interface with the flowing solar wind. The process of magnetic reconnection at Earth’s dayside magnetopause is recognized as the primary mechanism controlling the transfer of mass, momentum, and energy. This flow of energy in turn is what fuels geomagnetic storms within Earth’s magnetosphere, ionosphere, and thermosphere. If magnetic reconnection at the magnetopause is efficient and wide-spread, energy may transfer freely and cause the development of storms and disturbances. Over the past several decades a series of missions with decreasing inter-spacecraft spacing have been launched (ISEE-1/2 ( 1977), Cluster (Escoubet et al. 2001), THEMIS (Angelopoulos 2009), and MMS (Burch et al. 2016)) allowing progress in reconnection studies. These missions have provided illuminating breakthroughs on small-scale physics (electron and kinetic (tens of km)), however the community’s ability to understanding the macroscale (magnetohydrodynamic (MHD) and larger) or “cross-scale” physics remains a limitation in reconnection and solar wind-magnetosphere research. Since the macroscale solar wind-magnetosphere interaction controls the net flow of energy into the geospace system, this gap in knowledge leads to missing elements in the system energy budget.

Reconnection is linked to Earth’s magnetopause position through current systems, primarily region 1 Birkeland currents (Maltsev and Lyatsky 1975) and cross-tail currents (Wiltberger et al. 2003). As dayside reconnection occurs, these current systems are driven, which in turn decrease the dayside magnetospheric field strength. A decrease in magnetic field strength reduces the magnetic pressure and results in an Earth-ward motion of the magnetopause boundary. This link between reconnection and magnetopause position is captured in magnetopause models through inclusion of a parameter to quantify reconnection (Petrinec and Russell 1993; Shue et al. 1998; Lin et al. 2010) and reproduced in numerical models (Elsen and Winglee 1997; Xu et al. 2022). During periods of relatively stable dynamic pressure in the solar wind, magnetopause reconnection is the primary driver of the boundary position. Through tracking the boundary, one can study the dynamics of this global reconnection. To monitor this large-scale motion either global imaging or large constellations of spacecraft are required.

LEXI addresses macroscale features of reconnection at Earth’s dayside magnetopause. LEXI will probe the temporal stability of magnetic reconnection at the dayside magnetopause by targeting the question, *under what conditions is macroscale magnetic reconnection temporally stable versus variable?* This question was raised with some of the very first studies of reconnection in the 1970s (Haerendel et al. 1978; Paschmann et al. 1978; Russell and Elphic 1978) and continues to be a major topic of study without consensus. Over the years research has provided evidence for different temporal modes of reconnection.

#### Temporally Variable Reconnection

On one side of the discussion, evidence has been proposed for a temporally variable reconnection process. In this model, reconnection occurs in an impulsive, bursty fashion. Included in this model is a process which occurs continuously, but at a highly variable rate (Rosenqvist et al. 2008). If large-scale reconnection efficiency is modulated as a function of time, charged solar wind particles following the newly opened magnetic field lines will precipitate into the atmosphere with temporally variable auroral signatures at the magnetospheric cusp footprint. These have been observed through ground-based auroral imagers and presented as evidence of bursty reconnection (Oksavik et al. 2005; Davies et al. 2002; Lockwood et al. 2005).

Studies based on in-situ spacecraft measurements have also found reconnection to be intrinsically bursty, even during times with steady inflow parameters (Le et al. 1993; Zou et al. 2022). One magnetic signature of reconnection, Flux Transfer Events (FTEs) are commonly observed along the dayside magnetopause boundary. Temporally variable reconnection is commonly invoked to describe the formation of FTEs (Russell and Elphic 1978; Scholer 1988; Ku and Sibeck 1998). These features are typically understood in terms of very rapid bursts of reconnection separated by 7-10 minute lulls (Lockwood and Wild 1993; Wang et al. 2005). FTEs have scales sizes of 1-2 Earth radii ($R_{E}$) normal to the magnetopause (Rijnbeek et al. 1984; Fear et al. 2007).

Numerical modeling also shows evidence for temporally variable reconnection. Hybrid kinetic modeling with steady input solar wind conditions show highly dynamic magnetopause reconnection. In these models, reconnection occurs in an intrinsically dynamic fashion (Sibeck and Omidi 2012; Hoilijoki et al. 2017; Pfau-Kempf et al. 2020).

As a burst of reconnection occurs at the dayside magnetopause, a bundle of magnetic flux is removed from the dayside magnetosphere. As the bundle of flux is removed, the magnetopause boundary will leap inward with each burst. This motional signature of inward leaps can be targeted by magnetopause imaging. Some studies have shown a single FTE can add more than 10$\%$ of the total magnetic flux to the polar cap (Milan et al. 2000; Fear et al. 2017). Such a large and rapid depletion of dayside magnetosperic magnetic flux would have a significant impact on the magnetopause position.

**Fig. 2:**
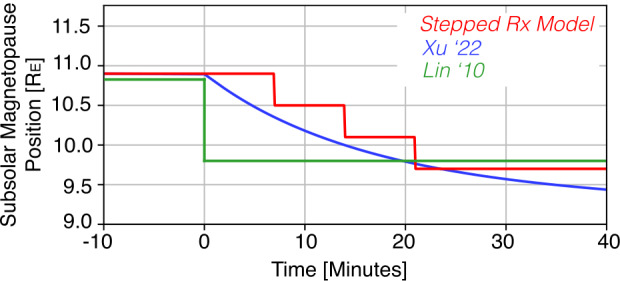
Comparison of predictions for the time-evolution of the dayside magnetopause. Solar wind undergoes a magnetic rotation at t=0 minutes from *Bz* = +5 nT to *Bz* = -5 nT with *Pdyn* = 2.0 nPa. Traces represent a stepped reconnection model (red), the Xu et al. (2022) model based on global MHD predictions (blue), and the Lin et al. (2010) magnetopause model

Through monitoring the position of the boundary, one can understand which mode of reconnection is dominant on a macroscale along Earth’s magnetopause boundary. Figure 2 compares proposed models of magnetic reconnection and the implications for magnetopause position during nominal driving conditions. Stepped or temporally transient reconnection, as is often associated with flux transfer events, manifests in inward leaps of the magnetopause boundary with a period of 7 minutes and steps of 0.4 $R_{E}$ (red trace). Although 7 minutes is the mean period, the distribution tail extends to longer periods with a median of 12 minutes (Wang et al. 2005). The magnitudes of the positions in Fig. 2 correspond to predictions from inputs of a steady dynamic pressure (*Pdyn* = 2.0 nPa) and magnetic field rotation of *Bz* = +5 nT to *Bz* = -5nT in GSM coordinates, initiating magnetic reconnection.

#### Temporally Stable Reconnection

By contrast, evidence has been presented to support a model where reconnection is temporally stable. Space-based measurements from the IMAGE spacecraft found a continuous cusp auroral footprint over the course of 3.5 hrs, indicating reconnection to be steadily and continuously occurring over a long period of time (Frey et al. 2003). Studies based on kinetic ion dispersions signatures in the cusps (Trattner et al. 1999) as well as at the magnetopause (Trattner et al. 2021) have also found evidence of temporally stable reconnection on time scales of tens of minutes up to an hour. Phan et al. (2004) found continuous detection of reconnection jets at the dayside magnetopause as the Cluster spacecraft encountered the boundary more than 10 times in a 2 hour window with steady IMF magnetic field and plasma conditions. This was presented as evidence for continuous reconnection.

Signatures of this reconnection model can also be monitored through macro-scale magnetopause imaging. If reconnection is occurring steadily at the magnetopause, magnetic flux will be steadily opened up as a function of time. As flux is opened, the magnetopause boundary will steadily move inward towards the Earth. This mode of global reconnection can therefore be probed through monitoring the location of the subsolar magnetopause. Figure 2 presents this motion as predicted through MHD modeling Xu et al. (2022) with the blue trace.

#### Boundary Condition Dependence

Lastly, the dominant temporal behavior of magnetic reconnection may be driven by the boundary conditions (Milan et al. 2016; Qudsi et al. 2023). Although the magnetic field orientation and plasma pressure on the magnetospheric side of the magnetopause are relatively constant (Tsyganenko and Sitnov 2007), the parameters in the magnetosheath vary significantly on time scales of minutes based on the driving solar wind (Dimmock et al. 2014). Spacecraft measurements of dayside magnetopause reconnection have found the process to occur in a quasi-periodic and pulsed nature with a distribution mean of 8 minutes. The same study found quasi-periodic variations in the magnetic field orientation in the solar wind with similar periods and proposed this changing boundary condition as a potential cause of the variations in reconnection (Lockwood and Wild 1993). Reconnection may also be suppressed by a gradient in plasma beta across the current sheet causing a diamagnetic drift (Swisdak et al. 2010). Along the dayside magnetopause this is primarily driven by plasma beta and magnetic field variations on the magnetosheath side of the boundary (Phan et al. 2013; Atz et al. 2022b). Lastly, work to quantify the rate of reconnection at the dayside magnetopause through a physics-based approach incorporates a number of parameters in the solar wind including density, velocity, magnetic field vector, and Alfvén Mach number (Borovsky 2013). Varying any of these parameters on short time scales would cause variability in reconnection in this and similar models (Lockwood and McWilliams 2021). Imaging the subsolar magnetopause position as a function of time in conjunction with solar wind measurements will provide necessary information to evaluate these proposed effects.

## Instrument Design

LEXI is designed to provide wide FOV soft X-ray images of the dayside magnetopause from the sun-lit lunar surface. A summary of the instrument parameters is provided in Table 1. The design has significant heritage from the STORM instrument (Collier et al. 2014, 2015) which flew as a technology demonstration on the Diffuse X-rays of the Local galaxy (DXL) sounding rocket in 2012 (Galeazzi et al. 2011; Thomas et al. 2013). The DXL mission provided observational evidence for the solar wind charge-exchange contribution to the soft X-ray galactic background (Galeazzi et al. 2014). Additional design heritage stems from the CuPID cubesat mission which flew a single optic X-ray imager (Walsh et al. 2021; Atz et al. 2022a). A similar tiled-MPO instrument has recently flown on the Lobster Eye Imager for Astronomy (LEIA) (Zhang et al. 2022) and will soon be carried on the Soft X-ray Imager (SXI) (Sembay et al. 2024) as part of the SMILE mission (Raab et al. 2016).

**Table 1:** LEXI instrument Parameters

Parameter	Value
Mass	11.1 kg
Power	1.6 W
Science Data Rate	40 kbps
Field of view	9.1^∘^ × 9.1^∘^
Dimensions	86 cm × 42 cm × 19 cm
Focal length	37.5 cm
Open Detector Area	44.18 cm^2^

### Science Requirements

To address the motivating science to understand the macroscale temporal response of magnetopause reconnection, LEXI will measure the subsolar magnetopause position as a function of time during a north to south rotation of the interplanetary magnetic field. This objective drives a number of requirements linked to the imaging capabilities of the payload including FOV, resolution, and throughput/image data extraction.

First, the telescope must have a sufficient FOV to capture the possible locations of the boundary. The telescope will slew its pointing slowly during course of the mission as the moon orbits Earth in order to maintain the average anticipated magnetopause position near the center of the FOV. This motion is relatively small, several degrees per day. Rather than attempting to articulate rapidly to respond to real-time solar wind conditions, the FOV is designed to be large enough to capture possible magnetopause motion for a range of solar wind conditions. This means the FOV must be large enough to capture the subsolar magnetopause boundary when it moves away from the average position. It must be able to capture the boundary during strong solar wind driving, or large dynamic pressure, when the boundary is compressed inwards towards Earth, as well as times with lower dynamic pressure solar wind when the boundary expands and moves radially outward from Earth. From a lunar vantage point, a FOV of 9.1$^{\circ}\times 9.1^{ \circ}$ allows imaging over a radial distance of roughly 15.2–6.1 $R_{E}$ from Earth’s center. During a similar time in the last solar cycle, the magnetopause was within these inner and outer bounds (in LEXI’s FOV) 99.99% of the time using the PRIME solar wind dataset (O’Brien et al. 2023).

Spatially, the magnetopause can make leaps of 0.4 $R_{E}$ during temporal burst of reconnection. Although 0.4 $R_{E}$ is a typical value, the amount of magnetic flux in a burst of reconnection can vary and motion can be larger or smaller (Fear et al. 2017). To capture this motion, LEXI is required to image with a spatial resolution of 0.2 $R_{E}$. From the lunar vantage point, this maps to an angular resolution requirement of 0.2^∘^. This drives the performance of the optics as well as the position-sensing detector described in Sect. 3.4. LEXI has an angular resolution of 0.2^∘^.

To differentiate between bursts of reconnection and steady erosion at the dayside magnetopause, the system is required to produce measurements every 5 minutes during a solar wind magnetic field rotation. This is defined by the experimentally measured periodicity of FTE at Earth. Since LEXI captures and telemeters to the ground the position of every photon sensed, the time resolution is a function of the count rate and the rate at which a sufficient image can be populated. The count rate is a function of two parts: (1) a fixed payload throughput, efficiency, and background, and (2) a time-variable solar wind flux. A time-variable solar wind flux results in the ability to generate sufficient images at different time cadences throughout the mission. Measured solar wind data from a similar time window during the previous solar cycle was used to bound this problem and determined an anticipated time resolution. Using the archival solar wind data, a 5 minute time resolution could be satisfied during 95% of possible operational windows. Further description of a data extraction process from LEXI images is presented in Sect. 7.

### Opto-Mechanical

**Fig. 3:**
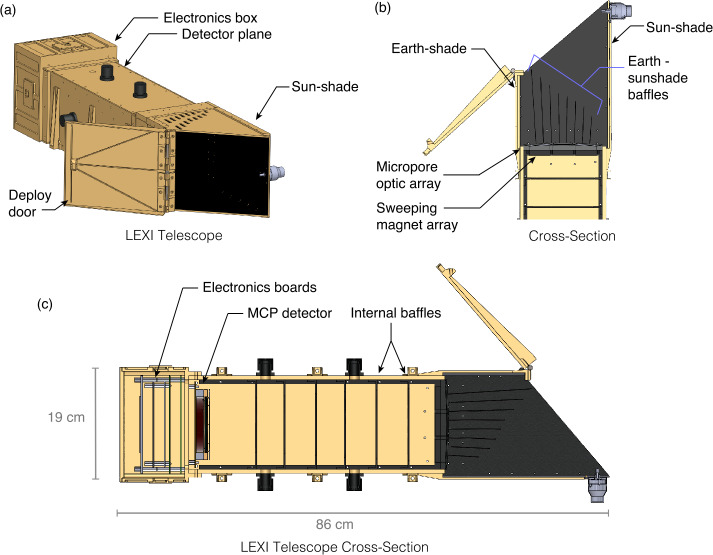
(a) Layout of the LEXI telescope. (b) A cross section of the Earth-sunshade and top of the telescope. The geometry shows the Earth-sunshade baffles alignment, the sweeping magnets, and internal blocking baffles. (c) Full telescope cross-section

LEXI is designed to collect and measure soft X-rays from the magnetosheath while rejecting incident charged particles and blocking light from the bright sun and Earth. Figure 3 presents a CAD model of the telescope. As a photon is incident on the telescope, the first element encountered is the Earth-sunshade. The Earth-sunshade is composed of a series of fins angled at the radius of curvature of 75 cm matching the optics.

**Fig. 4:**
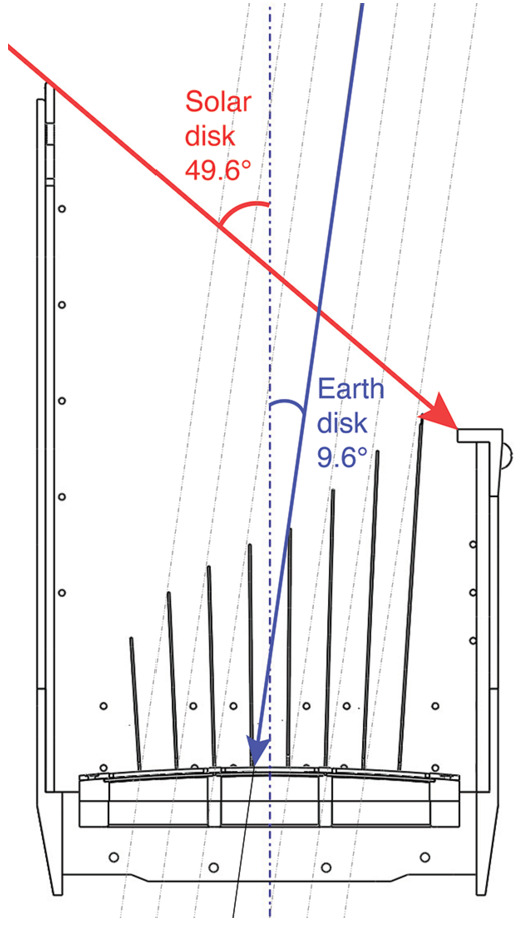
Sun-Earth shade geometry presenting the fin system and light blocking on the sun and Earth-side of the instrument. The labeled angles show the keep-out limits to viewing towards the sun (no closer than 49.6^∘^) and towards Earth (no closer than 9.6^∘^)

**Fig. 5:**
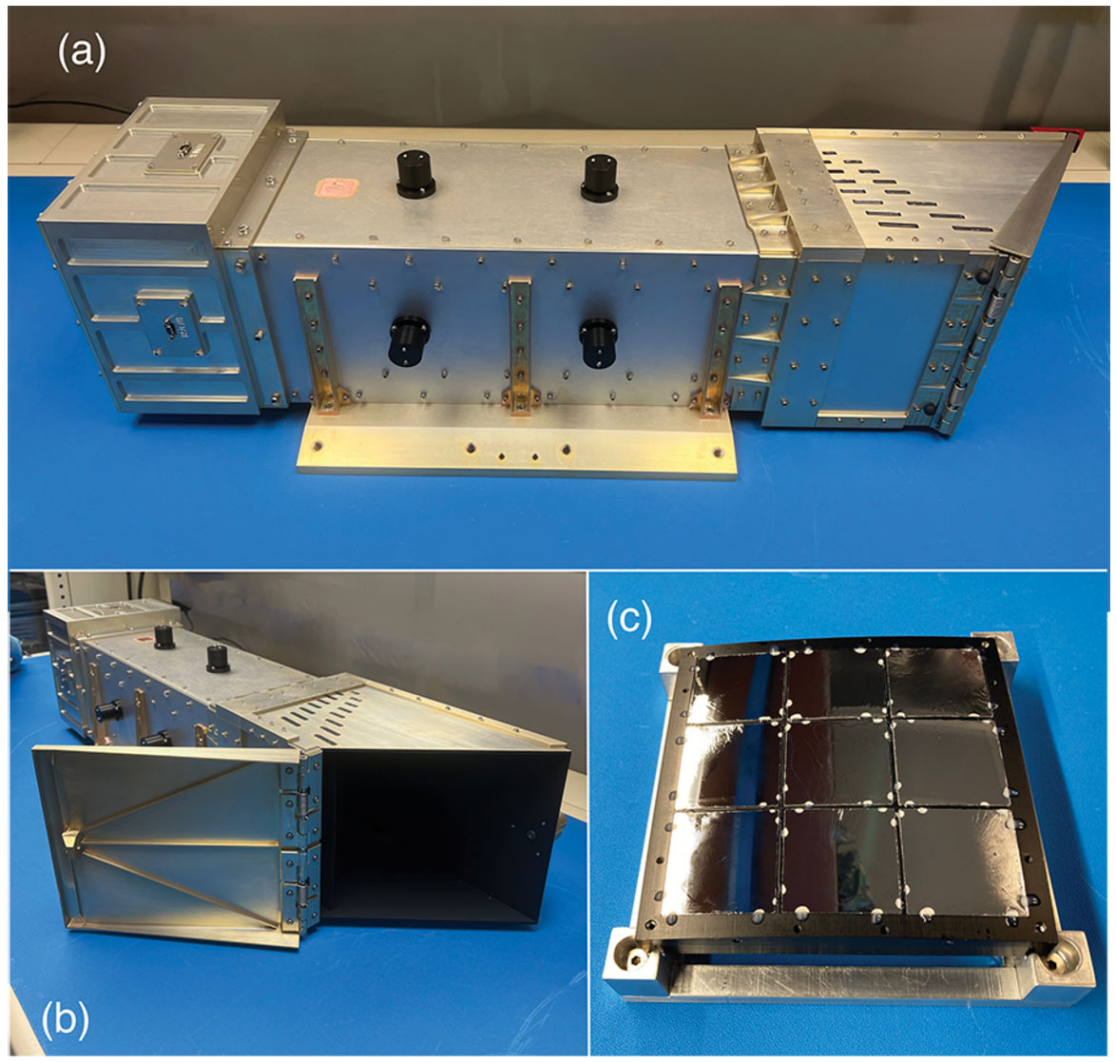
LEXI flight unit and components with the door closed (a) and open (b). Panel (c) presents the optic array before integration with the telescope body

Figure 4 further shows the geometry of the sun-Earth shade and the baffle or “fin” system. The structure blocks photons from angles up to 49.6^∘^ from the sun and 9.6^∘^ from Earth’s disk. The system shades itself completely from solar photons entering the aperture. On the Earth-side, the fin system prevents unwanted off-axis photons from directly encountering the optics without at least one bounce on the fins. As more fins are added, the length of the fins and sun-shade can be shortened. Although shorter fins allows for a more compact instrument, there is a penalty to throughput as each fin blocks a small percent of photons linked to the fins thickness. For the LEXI system, 8 evenly-spaced fins were adopted as a trade-off between size and throughput. A secondary benefit of the fins for stray light supression is that photons reflecting off their surfaces will be incident on the optics at large angles. Photons entering the optics at large angles (> a few degrees) have a significantly higher probability of being absorbed, with suppression as much as 5×10^−4^ (Sembay et al. 2024). Components internal to the sun and Earth shade are black painted to reduce reflectivity (<5$\%$ hemispheric reflectance) in the soft X-ray and UV. The flight unit is shown in Fig. 5 illustrating the sun and Earth shade design.

Once through the Earth-sunshade, an X-ray photon encounters the focusing optics. A series of nine tiled “lobster-eye” micropore optics (Fraser et al. 1992; Kaaret et al. 1992) form the focusing plane in a three by three array shown in Fig. 5c. To create the wide FOV the optics are mounted in an array on a spherical-sector metal frame with a radius of curvature matching the radius of curvature of the optics. Each 4 cm × 4 cm slumped micropore optical element is composed of an array of 20 $\mu $m × 20 $\mu $m square-hole micropores with 5 micron walls, for a 60% open fraction. The optical elements were manufactured by Photonis France SAS. For an imager with a fixed focal length, increasing the size of the optical plane expands the imaging FOV. In this case, the 3 × 3 array is sufficient to meet the science objectives. Similar tiled-optic systems have been used on BepiColumbo (Fraser et al. 2010) as well as Einstein Probe (Yuan et al. 2018).

**Fig. 6:**
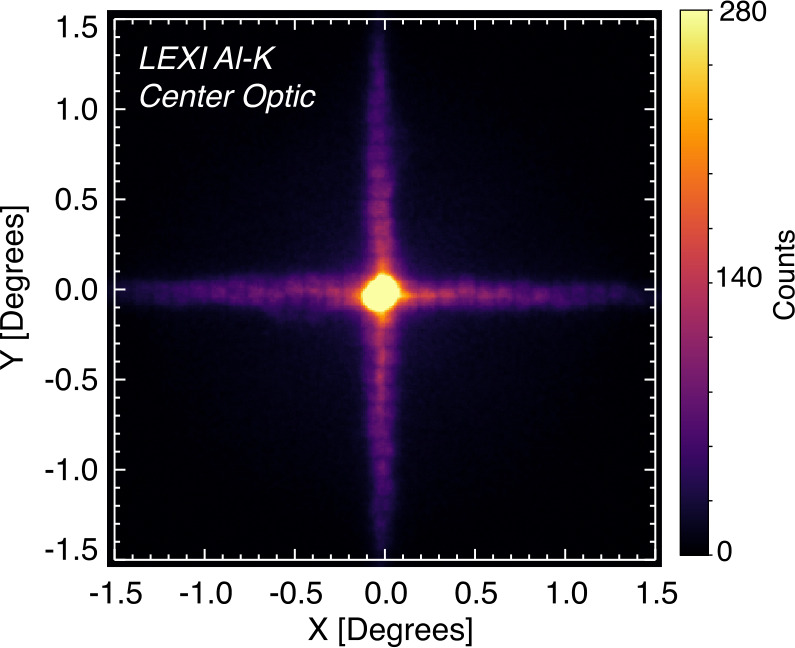
PSF of LEXI with a 1.487 keV photon beam incident on the center of the optical array. Counts are presented on a linear scale from a 60 s integration

To block unwanted UV and visible light, a 30 nm Al and 200 nm polyimide blocking filter is mounted on the convex side of each optic. The surfaces of the optic’s pores form reflecting surfaces orthogonal to the surface of the spherical optic, causing X-rays from infinity to focus to an image surface at half the sphere’s radius. The width of the pore to the length ratio (W/L) is $\frac{1}{50} $. Due to the geometry of the system, some fraction of photons pass directly through the pore without interacting with the walls. These photons remain undeviated in their path and unfocused. A photon that reflects off a single wall (with a single or an odd number of bounces off walls in a single dimension) will be focused in a single axis and form the cross-shaped PSF arms. A photon that makes reflections off two orthogonal walls (with a single or an odd number of bounces from walls in each dimension) will be focused in two dimensions and are fully focused to form the core of the point spread function (PSF) shown in Fig. 6.

The position sensing of the system is limited by the performance of the optics and the angular width of the PSF. With the calibration beam directed on the middle of the optical plane, the resulting PSF has a full-width half maximum (FWHM) of 12.43 arcmin or 0.20^∘^. The system has slightly different PSF when light is illuminating different areas of the optical plane (Paw U et al. 2022; Kuntz et al. 2023). Since the moon’s possible distance from Earth’s dayside magnetopause ranges from 57 to 64 $R_{E}$ during the operational window, the angular resolution of LEXI projected to the dayside magnetosphere is approximately 0.2 $R_{E}$. One should note that the spatial resolution is a function of the radial distance of the observer. With a fixed angular resolution, the spatial resolution becomes better (smaller) as an observer moves closer to the target. In this case, since the moon’s orbit is close to circular, and LEXI has a short observing period, this property does not have a significant impact on the LEXI resolution during planned observations. Further description of the telescope characterization testing is included in Sect. 3.4.

Below the optical plane is a sweeping magnet array to deflect charged particles. Mechanically, the magnet array has the same profile as the frame holding the MPO and thus does not impact the X-ray throughput. The fixed sweeping magnet array is composed of 48 neodymium magnets configured as a hexapole to maximize the local field strength while dropping off spatially to minimize impact on other payloads and spacecraft components. The DC field generated is greater than 10^6^ nT near the optics and decreases to a magnitude of less than 10 nT at a distance of 1 m and less than 0.1 nT at 2 m, satisfying the spacecraft magnetic requirement of a dipole moment less than 400 mAm^2^.

**Fig. 7:**
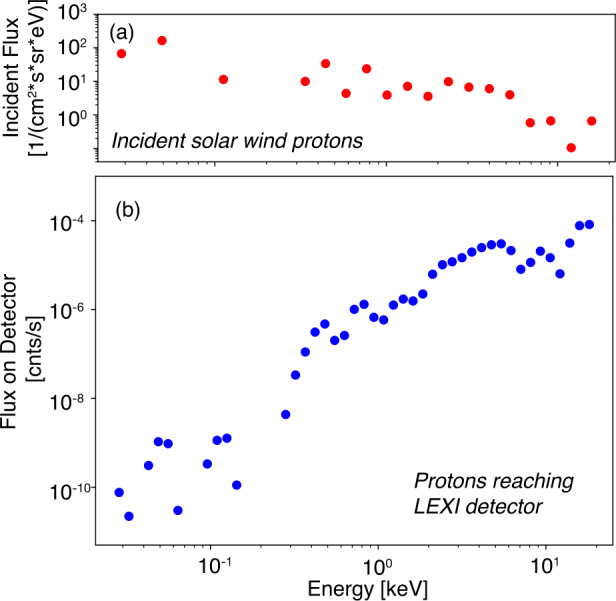
Charged particles at LEXI. (a) Proton flux measured by THEMIS at the moon in the solar wind. The flux is from a look direction corresponding to LEXI’s science pointing, 60^∘^-120^∘^ from the Earth-sun line. (b) Modeled solar wind protons reaching the LEXI detector after passing through the sweeping magnets and blocking filter. Total incident proton counts is modeled to be less than 0.5 s^−1^ for nominal solar wind conditions

For typical solar wind conditions, LEXI will be on the lunar surface within the solar wind during the entire science mission. From the reference frame of the lunar surface, the solar wind will also be highly directional with nominal bulk flows of 400 - 500 km/s in the anti-sunward direction. Since LEXI will always be pointing at least 49.6^∘^ away from the sun, the flux of incoming charged particles is further reduced. To quantify the anticipated charged particle background rate, the system was simulated using a fully kinetic particle tracer with a fifth order Runge-Kutta solver. The simulation used LEXI’s mechanical structure, sweeping magnetic field, filter particle transmission, and a measured particle distribution. The solar wind particle distribution at the moon was taken from the THEMIS mission (Angelopoulos 2009) and used as the input. Integrated over energy, the simulation found the total throughput to be less than 0.5 counts per second (Paw U et al. 2024). Figure 7 presents the incident flux from the solar wind protons as well as the anticipated counts on the detector after passing through the particle rejection system. Due to their smaller gyroradii, no incident electrons made it to the detector. Although some other Earth-orbiting X-ray missions have experienced appreciable particle backgrounds while regularly passing through regions of geomagnetically trapped energetic particles (>100 keV) (Snowden et al. 1992; O’Dell et al. 2007; Walsh et al. 2014), the combination of the sweeping magnet geometry and radiation environment at the moon allow for a high level of charged particle suppression for LEXI.

### Detector and Signal Chain

Once a photon reaches the detector plane it generates a signal and is recorded as an individual event in a 16 Byte packet. Each valid event is logged, passed to the spacecraft, and telemetered to the ground. It is important to emphasize that in this format, *there is no on-board time integration or exposure time*. Since each photon is logged, the researcher on the ground can pick appropriate time-integration or pixel shape(s) and sizes to best meet a given science-goal. This process is described in more detail in Sect. 7.

**Fig. 8:**
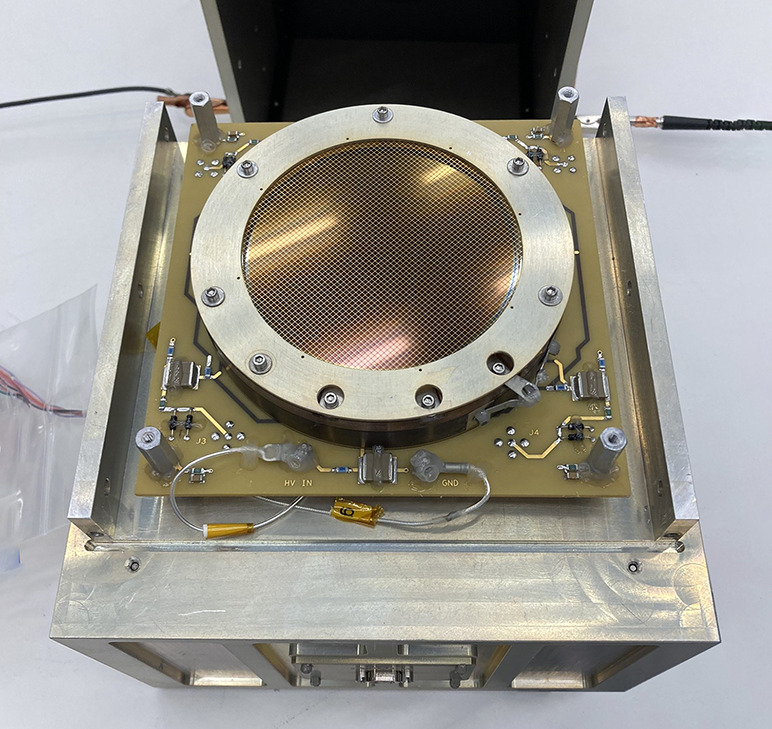
LEXI MCP detector and electronics box

The signal chain starts with a photon encountering the 80 mm diameter Potassium Bromide coated Micro Channel Plates (KBr MCPs) in a chevron configuration with 25 $\mu $m pores. Part of the MCP is blocked mechanically for background monitoring and the diameter of open area is 75 mm. Figure 8 presents an photograph of the detector mounted on the electronic box in flight configuration. As the photon encounters the pores of the MCP, an electron cloud is generated which is swept to a position-sensing wedge and strip anode with ∼ +2,100 V bias across the MCP. The process of conversion of the incident photon to the electron cloud has a gain of ∼ 10^5^ in the process. Four voltages are produced by the wedge and strip anode. The voltages are then passed to Amptek A111 preamplifiers then peak-hold circuit that holds the maximum amplitude of the raw signal pulses for roughly 8 $\mu $s and provides a trigger pulse to initiate digitizing the signals. The position of each photon strike is determined through a ratio of the voltages in each the wedge and strip directions.

The detector position sensing capability was measured through stimulating regions of the detector with an Al mask over the sensing area. The position sensing resolution was found to be 0.65 mm on the detector which corresponds to 0.07^∘^. Since the optics PSF core is 0.2^∘^, the system is therefore limited by the optics and the optical resolution of the telescope is 0.2^∘^ (root-sum-square of optic and detector resolution) in regions of the focusing plane that will be used in science imaging. Aberrations occurring in the corners of the FOV increase the angular resolution to 0.3^∘^.

The analog voltage signals corresponding to each detection event are passed to a Command and Data Handling (C&DH) board which processes the signal and manages instrument operation. The analog signals are converted to digital with a 16 bit successive-approximation Analog to Digital Converter (ADC) and packetized. The ProAsic3E field-programmable gate array (FPGA) appends mission-elapsed time tags to each packet to the nearest millisecond. Additional time tags are added to the packets later by the spacecraft which calibrates their clock using GPS and telemetry signals sent from the ground at Earth. Adjustable minimum and maximum pulse height voltage thresholds are set in the FPGA. These are used to allow the instrument to throw away events detected by the MCP which are not thought to be caused by incident X-ray photons. The C&DH also controls the primary and backup deploy circuits for the deploy door.

**Fig. 9:**
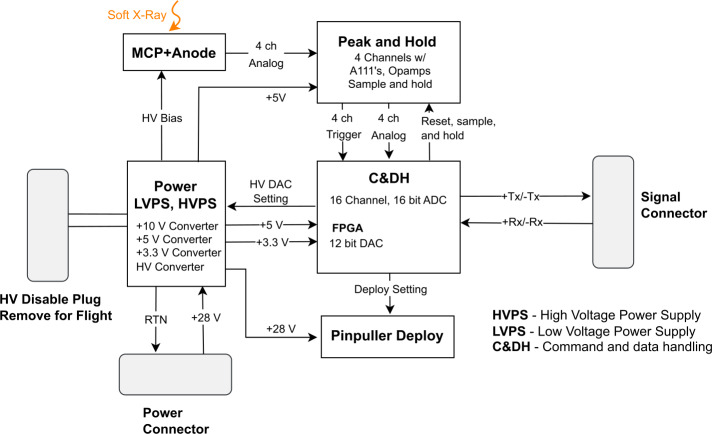
Functional block diagram of the LEXI signal chain

The system is powered with a single instrument power board which regulates the incoming +28 V from the spacecraft and generates both the necessary high and low voltages for the payload. A block diagram of the full signal chain is presented in Fig. 9. The system takes 14 $\mu $s to process a single pulse internally, however the payload is ultimately limited by the communication protocol with the spacecraft which sets a maximum rate on incoming photons as 2,880 events/s. The expected signal is fewer than 400 counts/s. Spatially, the detector and signal chain electronics are in a single stack on the end of the payload opposite the aperture (as shown in Fig. 3c).

### Instrument Performance

After component-level characterization and assembly, integrated instrument performance was measured through several test campaigns including beam line experiments at the PANTER facility (Bradshaw et al. 2019) in Germany and the Stray-Light Facility (Thomas and Baumgartner 2022) at NASA’s Marshall Space Flight Center (MSFC) in the United States.

#### Optical Calibration

LEXI optic calibration was conducted at the PANTER facility in Neuried, Germany. PANTER is a facility of the Max-Planck-Institut für extraterrestrische Physik in Garching. The PANTER vacuum beamline is 120 m long, with a further 12 m long chamber to minimize divergence (859 arcsecs). Two different CCD detectors were used to characterize the optic while a silicon drift detector (SDD) was used to monitor the beam stability. The intensity was measured to be stable within 2% during testing. The beam itself is provided by a well characterized electron impact source with one of a number of targets.

Optical calibration focused on performance of the focal plane, and the optic array was tested independently in the beamline with the reference detectors. The focal length and point spread function (PSF) were characterized through testing with a continuum emission as well discrete energy lines (B-K, 0.183 keV; C-K, 0.277 keV; Ti-L, 0.452 keV; O-K, 0.525 keV; Cu-L, 0.929 keV; Al-K, 1.487 keV; Ag-L, 2.98 keV) ranging over the target energy range. Figure 6 presents the PSF of the center optical facet for Al-K (1.487 keV). The emission line was selected for this characterization due to its brightness. The core of the PSF centroid was fit to a hyperbola and has a FWHM of 12.43 arcmin or 0.2^∘^. The periodic dark lines roughly every 13 arcmin in the arms of the PSF are a manufacturing artifact caused by the MPO being composed of sub-bundles of pores.

**Fig. 10:**
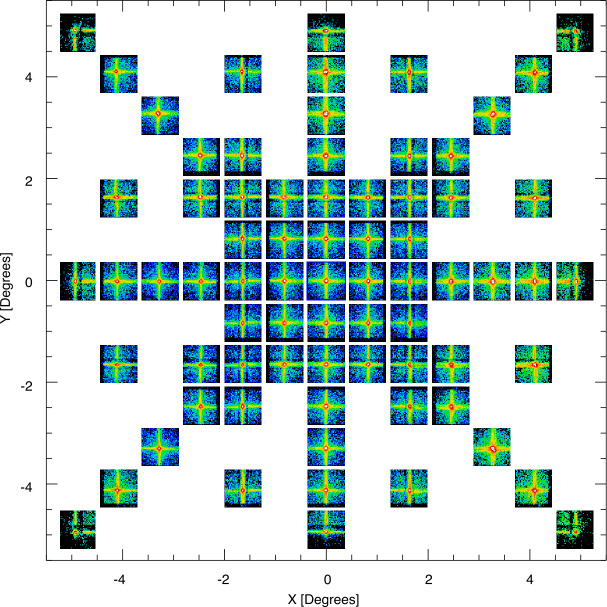
Focal plane mapping of the LEXI FOV at O-K (0.525 keV). The mosaic of 600 s images shows the focusing performance as the X-ray beam was scanned over the focal plane

The performance of the full focal plane was mapped through articulating the optic array so the X-ray beam was pointed at different points both on-axis and off-axis. A compilation of 600 s images from the O-K line at 0.525 keV is presented in Fig. 10. The scanning pattern was designed to maximize observations near the center of the optic array where the target is placed. Efficient focusing is shown over the full array spanning each of the nine optical facets. Some artifacts are visible when the beam is incident on the mechanical structure supporting the optics. These appear as horizontal and vertical bands and slightly lower throughput at ±∼1.5^∘^ from the center of the FOV. The size of the PSF core FWHM also has some variation over the FOV where the PSF is wider near the edges of the FOV by roughly 3 arcmin (0.05^∘^) compared to the center. This feature is primarily driven by the property that the detector plane is flat while the focal plane is hemispherical. The result is that points on the detector are increasingly out-of-focus towards the edges of the detector plane. The extreme of this effect occurs in the corners of the FOV where the PSF reaches a maximum value of 0.3^∘^, however it is not anticipated for this region of the optic array to be centered on the primary science target during the mission. Further description of the LEXI calibration at PANTER is provided by (Paw U et al. 2022; Kuntz et al. 2023).

#### Instrument Calibration

Full instrument X-ray calibration was conducted at MSFC’s Stray-Light Facility in Huntsville, Alabama. The 104 m vacuum beam line provided low divergence incident photons at several energies (C-K, 0.277 keV; Ti-L, 0.452 keV; Al-K, 1.487 keV). Beam stability and intensity were monitored with a calibrated SDD. The test campaign at MSFC included the full integrated payload in flight configuration.

**Fig. 11:**
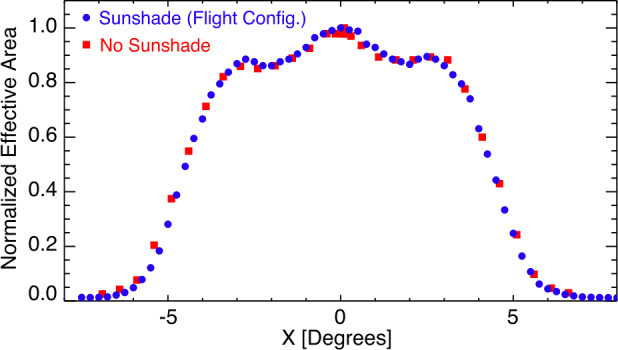
Relative efficiency of LEXI over the FOV at 1.487 keV with 0.25^∘^ steps with (blue) and without (red) the Earth-sunshade installed on the telescope. Local decreases in throughput at ± ∼ 1.5^∘^ from the center of the FOV are a result of the mechanical structure supporting the tiled optics

One feature of lobster-eye optics versus alternative X-ray focusing systems such as Wolter I is that lobster-eye optics offer a relatively constant effective area over much of the FOV. Figure 11 presents the vignetting function or the response of the telescope as a function of incident angle with and without the Earth-sunshade installed. The similar trends demonstrate the angled Earth-sunshade fins do not block appreciable amounts of target photons. The eight Earth-sunshade fins do not present clear signatures in the vignetting. In both cases, at large off-axis angles, the efficiency of X-ray reflections in the optics decreases, which decreases the effective area near the edges of the FOV. The two dips at ∼ ±1.5^∘^ from the center of the FOV are the result of the mechanical support structure holding the MPO. Since the mechanical optic support is symmetric in the X and Y axis, the vignetting function is the same in both axis. The effects of this structure can also be seen in Fig. 10.

## Backgrounds

Several backgrounds must be characterized and removed in order to isolate the X-ray emission from Earth’s magnetosheath including the cosmic X-ray background, the near-Earth Extreme Ultraviolet (EUV) emission, and instrument background.

### Soft X-ray and UV

**Fig. 12:**
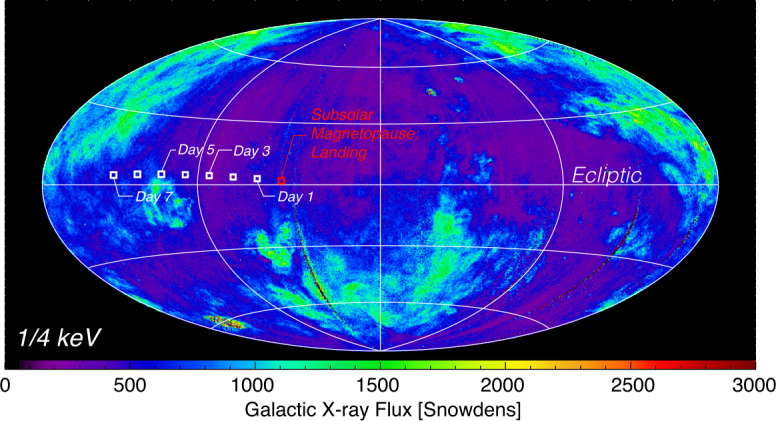
The galactic soft X-ray background in the $\frac{1}{4} $ keV (R12) energy band from ROSAT. This band includes with the bandpass of LEXI. Flux is presented in ecliptic coordinates ($\lambda $, $\beta $) where the horizontal axis is the ecliptic plane and $\lambda $=0^∘^ is at the center. Flux is presented in “Snowdens” or 10^−6^ counts s^−1^ arcmin^−2^. The squares represent the look direction of the science target from LEXI on the lunar surface with one day cadence for the planned operations period

The cosmic X-ray background varies strongly across the sky and has been measured accurately in the $\frac{1}{4} $ keV band by the ROSAT All-Sky Survey (RASS, see Fig. 12) (Trümper 1982) which spans the LEXI energy band. Points are plotted at the location of the science target in the sky as observed from LEXI on the lunar surface during operations. Although the background is spatially non-uniform, it varies on a range of time-scales all significantly longer than the geocoronal solar wind charge-exchange component observed by LEXI. This means the background remains relatively static temporally and can be removed from magnetosheath X-ray emission which varies on time-scales of minutes. As the ROSAT response is not the same as the LEXI response, the RASS values must be scaled to LEXI using the spectrum of the emission and the LEXI response. Over most of the sky the spectrum is well described by Kuntz and Snowden (2000), but there are large structures, such as the North Polar Spur, the Monogem supernova remnant, the Cygnus superbubble, etc., that must be handled differently. The team applies the techniques of Kuntz and Snowden (2000), with updated spectral models, and a more contemporary understanding of the heliospheric X-ray emission (e.g. Galeazzi et al. 2014) to produce a map of the spectrum of the cosmic X-ray background which can then be converted into a map of the background as seen by LEXI.

UV background for X-ray telescopes typically includes He^+^ 30.4 nm (40.8 eV) emission from Earth’s plasmasphere, H Ly-$\alpha $ emission (121.6 nm, 10.2 eV) scattered from the exosphere (Chakrabarti et al. 1982; Rairden et al. 1986; Sandel et al. 2001), as well as interplanetary emission (Baliukin et al. 2019). With LEXI’s pointing geometry and avoidance angles from Earth’s disk, the plasmasphere will not be within the FOV. The Ly-$\alpha $ signal from the exosphere decreases with distance from Earth as the exosphere density decreases. The Ly-$\alpha $ signal from the magnetosheath and outer-magnetosphere as well as interplanetary source (several hundred Rayleigh up to 1 kRayleigh) will be attenuated significantly with the UV blocking filter mounted on the LEXI optics (∼10^−6^). The team uses the solar irradiance and the spatial distribution of H and He^+^ to model this foreground emission following previous work by Snowden and Freyberg (1993). Flexible software tools to remove these UV and X-ray backgrounds for a variety of missions have been developed by Johns Hopkins University and are employed to clean the data.

### Detector

**Fig. 13:**
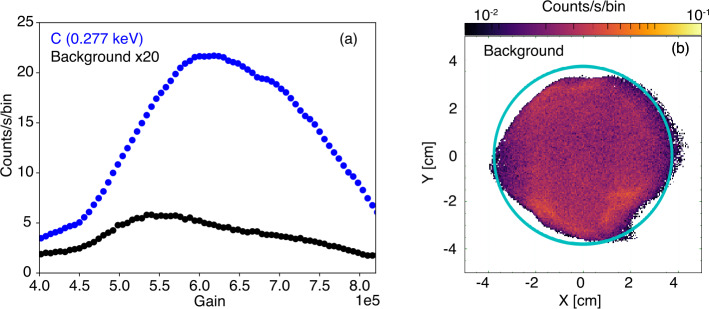
(a) MCP pulse height distributions for background (black) and C-K (0.277 keV) signal (blue) with +2,100 V across the MCP. (b) Distribution of instrument background over the detector

Instrument background was characterized through monitoring pulse height distributions and dark images from the MCP detector. The background level was measured to be 0.40 - 0.44 events s^−1^ cm^−2^ or 18 - 22 events s^−1^ over the detector, similar to previous missions with flat MCP imaging systems (Sandel et al. 2000; Siegmund et al. 2016). The MCP system is robust thermally and the background variation during thermal vacuum testing over the operational temperature range is on the order of 1$\%$. These detector backgrounds are primarily due to $\beta $-decay from ^40^K in the MCP glass (Siegmund et al. 1988). From this mechanism these background events are generated and detected throughout the bulk of the glass, thus producing a broad distribution decreasing pulse amplitude distribution with gain as compared to the peaked soft X-ray photon event pulse amplitude distributions (shown in Fig. 13a). Spatially, the detector background is nearly uniform over the exposed area as shown in Fig. 13b. This presents the background accumulated during a continuous 24 hour dark image with +2,100 V across the detector.

## Lunar Surface Operations

Primary science observations will occur from the lunar surface. LEXI is mounted on a pointing gimbal on the top deck of the lander roughly 2 meters from the lunar surface. The spacecraft will land on the surface shortly after sunrise on Mare Crisium. During landing the optics are protected by a mechanical door on the payload held closed by a TiNi actuator. After landing, the actuator is deployed through a 1.5 A current pulse and the door is opened by torsional springs to expose the telescope’s aperture. Instrument-level testing of the payload in a landing-generated dust cloud was evaluated through a series of test runs in a dust settling chamber. Focused testing on the door and hinge were conducted using BP-1 lunar dust simulant (Stoeser et al. 2010; Suescun-Florez et al. 2015) to verify sufficiently low friction on moving parts. The spring system provides 0.17 N ⋅ m of torque to open the door. Although some bodies have winds or dust storms which can cause dust accumulation, this is not expected on the lunar surface. Appreciable dust accumulation after landing over the time period of the mission is not anticipated.

**Fig. 14:**
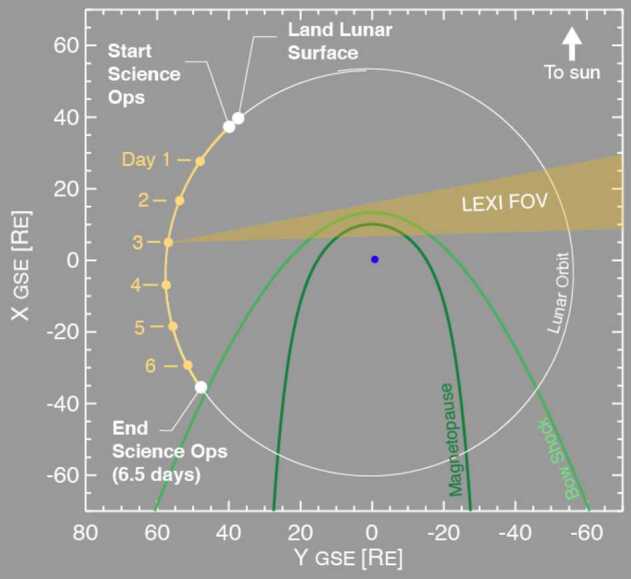
Operations geometry from the lunar surface. The diagram is to scale with Earth in the center in GSE coordinates. The 6.5 day operation window is highlighted in yellow while the look direction articulates to capture the magnetosheath and magnetopause

Once the door is open, LEXI will point at the dayside magnetopause. Pointing will slew to track the magnetopause while the moon orbits around Earth, slowly changing the view angle. Figure 14 presents the science pointing geometry during the mission. LEXI’s 9.1^∘^x 9.1^∘^ FOV allows for capture of the magnetopause position as it compresses and relaxes in response to variations in the solar wind without responsive pointing. For nominal solar wind variability, the subsolar point can move 2-3 $R_{E}$ (Sibeck and Gosling 1996; Haaland et al. 2004; Plaschke et al. 2009) or roughly 2^∘^-3^∘^ in the sky from the lunar platform. The science mission will last roughly 6.5 days and will end as the lunar phase approaches full moon, when the angle between the sun and Earth is too small in the sky to maintain the angular sun and Earth keep-out conditions (49.6^∘^ and 9.6^∘^ respectively). As appropriate for a mission short in duration, science data will be telemetered to the team on Earth within 24 hours of collection. The lander is not designed to survive the extreme cold of lunar night. Throughout the operational period the payload will be thermally controlled to remain between -10^∘^ and +50 ^∘^C. This means LEXI is not anticipated to perform additional science operations when a similar observing geometry occurs again roughly 28 days from the start of the first period. The mission is designed to land at Mare Crisium at a specific local time on the lunar surface, near local sunrise. If a launch or landing window is missed, a new one will open roughly 28 days later after one lunar orbit around Earth.

The operational modes for the payload include Off, Standby, and Imaging. Off represents a setting with no power provided to the payload. In Standby mode the payload is receiving power from the spacecraft and LEXI provides housekeeping data at 1 Hz containing current and voltage monitors, mission elapsed time, configuration settings, and temperatures. When commanded to Imaging mode, high voltage is set on the MCP and X-ray counts are measured through the system and transmitted in telemetry. Due to the short science observing window for the mission, LEXI is commanded to Imaging mode less than 12 hours after landing on the lunar surface. Over the course of the mission the payload electronics are anticipated to be exposed to 0.3 krad of radiation with the majority of that coming during transit between Earth and the moon.

## Data Pipeline

Since every photon event is transmitted to the ground, the data products can be calibrated and cleaned through ground-processing. Table 2 presents the mission’s data products. In contrast to some other imaging instruments used in heliophysics, the primary data product for LEXI is photon counts or a time-tagged “photon event list.” Data remains in this format to maximize flexibility for science analysis. It allows the user to select a user-defined time-integration period or pixel size and shape for the science application. In general, time periods with higher solar wind flux will permit higher time resolution (shorter time integration) images and/or smaller pixels.

**Table 2:** LEXI Data products

Data Level		Description
Level 0		Binary data from spacecraft
Level 1	a	Time, voltage channels from the detector, and housekeeping
	b	Time, corrected x,y spatial positioning of photon events in detector coordinate system
Level 2	a	Time, angular position of photon events (J2000, RA, DEC coordinates), lunar ephemeris/observer location, boresight pointing
	b	Background-corrected images with fixed pixel sizes

Within Level 1, photon counts or “events” are presented in telescope coordinates and corrected for offsets in detector spatial position and non-linearity effects quantified through masked pin-hole images taken during ground calibration (Level 1b), similar to other MCP imaging systems (Sandel et al. 2000; Zhang et al. 2018). The offsets are made to set the center of the boresight as [0,0]. The transition from Level 1b to Level 2a moves from telescope coordinates to physical angular units in the sky. This transformation is made using a time series of pointing vectors provided by the lander. The telescope pointing vector and roll angle are provided in Level 2a to allow the generation of an exposure map for a user-defined integration time period and pixel size. Level 2b will provide background-corrected images for a fixed integration period and pixel size. Level 2a and 2b data can be used for scientific analysis.

Data will be stored and made publicly available in the Space Physics Data Facility (SPDF) Coordinated Data Analysis Web (CDAWeb) at NASA Goddard Space Flight Center.

## Science Analysis

**Fig. 15:**
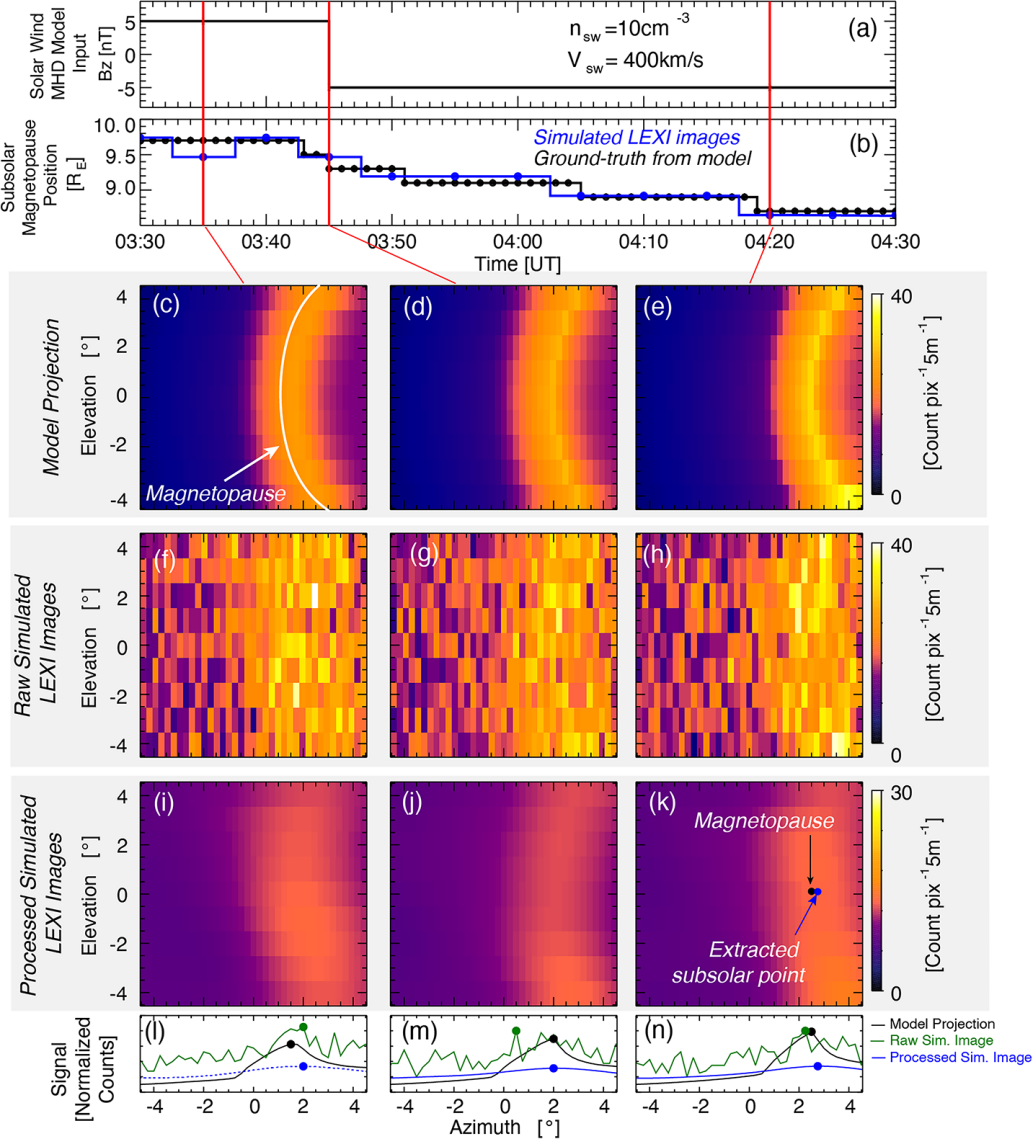
Demonstration of magnetopause boundary retrieval from simulated soft X-ray images. Images include relevant throughput and background sources for a 5 minute integration from LEXI. The magnetopause is highlighted in panel (c) for demonstration. The blue circle in panels i-k represent the derived subsolar magnetopuse position. Panels l-m, show the count profiles along the Earth-sun line

Soft X-ray emissions collected by LEXI are used to probe the position of the dayside magnetopause to target its motivating science questions. Since the detection of each X-ray will be transmitted to the ground in an event list, a user has the ability to integrate for short or long time periods with user-defined pixel sizes and shapes to match the necessary time-cadences and signal-to-noise required for a particular science question. In general as one uses shorter time cadences, larger pixels are required to maintain a constant signal-to-noise. Simulated images from LEXI during a solar wind magnetic field rotation are presented in Fig. 15. The simulation process follows that used by Connor et al. (2021). Images are produced using (1) a numerical global MHD model to provide plasma parameters, (2) an analytical geocorona model to provide neutral densities, (3) an empirical model to provide a solar wind charge-exchange emission from the plasma and neutral densities as well as collisional speeds, (4) galactic soft x-ray backgrounds from ROSAT, (5) the instrument response as determined through payload calibration, and lastly (6) poisson noise.

In the MHD simulation, the input solar wind velocity and proton density are held constant at 400 km/s and 10 cm^−3^ respectively. The magnetic field starts at GSM *Bz* = +5 nT then rotates to *Bz* = -5 nT at 3:45 (HH:MM) in simulation time. Such a rotation is known to initiate or enhance magnetic reconnection at the dayside magnetopause which erodes the boundary inward toward Earth. This inward erosion is shown in the MHD simulation (15 c-e) as well the reconstructed images in the bottom row (15 i-k). The three columns are images from three different time steps during the 1 hr period shown in the Figure. Panel b presents the “ground-truth” position of the subsolar magnetopause from the MHD model (black line, panel b) as well as the extracted magnetopause boundary positions from the processed 5 minute-integrated LEXI images (blue line, panel b).

One useful method of image processing is also illustrated in Fig. 15. The raw integrated images from LEXI presented in Panels f-h show noise superimposed on the magnetospheric charge-exchange signal. Since the magnetospheric boundaries are anticipated to be large spatial structures in the images, one way to obtain useful images, with relatively short time-cadences and without a large density enhancement in the solar wind, is to use low pass filtering. Here a gaussian low pass filter is used to clean images from the middle row of Fig. 15 to the bottom row following a technique further described by (Kim et al. 2024). Within a slice of the magnetosphere in the noon-meridional plane, the magnetopause will typically appear as a relatively sharp transition in density or solar wind flux diving the dense magnetosheath from the tenuous magnetosphere. Due to projection effects however, when viewing as a ling-of-sight through the magnetosheath from the perspective of the moon, the azimuthal angle of peak flux corresponds to a line tangent the dayside subsolar magnetopause (Collier and Connor 2018; Sun et al. 2020; Samsonov et al. 2022). This method is often referred to as the tangent fitting approach (TFA). In Fig. 15b, each extracted point from the simulated image is within 0.2 $R_{E}$ (range from 0.0 - 0.2 $R_{E}$) of the Ground-truth position from the numerical simulation before noise and the instrument response has been added (white trace in panel c). This full-chain example demonstrates the ability to satisfy the motivating science to image the boundary with a spatial resolution of 0.2 $R_{E}$. This set of simulated observations shows gradual boundary motion over 40 minutes in response to a rapid IMF rotation and would be an example of a period of continuous reconnection at the magnetopause boundary.

Although an application of low pass filtering and the tangent fitting approach has been presented here, a number of other methods have been proposed to extract the magnetopause position and dynamics using soft X-ray images. One technique modeled with good success is using 2D X-ray images from a variety of vantage points with computed tomography (CT) to produce a 3D information of the boundary (Jorgensen et al. 2022; Wang et al. 2023). Such reconstructions are possible with LEXI but would require long time scales (days) to obtain the necessary range of vantage points (Cucho-Padin et al. 2024). Modifying the pixel shape and size has also been conducted and shown promising results (Sibeck et al. 2018).

An important component of these simulations is the driving solar wind conditions. Since the soft X-ray flux scales with solar wind flux (Kuntz et al. 2015), a period with higher solar wind density and velocity would produce higher counts and signal to noise. The opposite is also true, the counts are anticipated to be lower during periods of low solar wind flux.

## Additional Targets

Soft X-ray imaging has the potential to provide images illuminating a number of important magnetospheric dynamics (Walsh et al. 2016b; Sibeck et al. 2018). LEXI is designed to focus on the subsolar magnetopause, however other instrument designs are possible to capture different features where there is a strong gradient in solar wind density. Several possible targets are the magnetopause at positions outside the subsolar point, the cusps, and the bow shock. The SXI instrument on the SMILE mission is expected to provide images of the cusps from a variety of vantage points (Sembay et al. 2024; Carter et al. 2024). The pointing gimbal on the Blue Ghost lander prevents LEXI from viewing at large angles from the Earth-sun line and thus the payload is not anticipated to be able to see the cusps within the FOV unless there is extreme solar wind driving.

Regions of the magnetopause away from the subsolar point (∼ ± 4.5 $R_{E}$ from Earth-sun line) will regularly be in the LEXI dataset and could be probed to monitor the shape of the boundary as a function of time and solar wind driving. Models predict the magnetosphere to become more blunt in shape in response to strong solar wind driving which in turn modifies flow in the magnetosheath. Images of the boundary shape could study these effects. Looking forward, other future missions such as the Line Emission Mapper (LEM) concept offer extremely high energy resolution spectral measurements through the magnetospheath, providing valuable information on charge states and abundances of minor solar wind species (Küntz et al. 2024).

Since Earth’s bow shock resides in a region of lower exospheric neutral density than the magnetopause, and has a smaller density gradient than at the magnetopause, the charge-exchange X-ray signal is smaller. An imager such as LEXI scales well, and a payload with a larger effective area could be optimized to study this boundary through charge-exchange emissions. Although the position of Earth’s bow shock is likely to be in the FOV, it is not anticipated the position will be observable during most solar wind driving conditions. Missions with larger telescopes such as the STORM concept (Sibeck et al. 2023) may be able to probe these boundaries.

## Summary

LEXI will provide high-quality position sensing measurements of soft X-rays (0.1–2 keV) from Earth’s magnetosheath. LEXI will conduct science operations for roughly 6.5 days from the near-side lunar surface on a lunar lander. During the operational period the telescope will continuously image Earth’s magnetosheath and dayside magnetopause boundary with a FOV of 9.1$^{ \circ}\times 9.1^{\circ}$. LEXI will be able to monitor the subsolar magnetopause position with a variety of solar wind driving parameters to monitor its motion as a function of time and driving condition.
